# The Small Ethylene Response Factor ERF96 is Involved in the Regulation of the Abscisic Acid Response in *Arabidopsis*

**DOI:** 10.3389/fpls.2015.01064

**Published:** 2015-11-26

**Authors:** Xiaoping Wang, Shanda Liu, Hainan Tian, Shucai Wang, Jin-Gui Chen

**Affiliations:** ^1^Key Laboratory of Molecular Epigenetics of Ministry of Education and Institute of Genetics and Cytology, Northeast Normal University, Changchun, China; ^2^Biosciences Division, Oak Ridge National Laboratory, Oak Ridge, TN, USA

**Keywords:** ERF96, ethylene response factor, transcription factor, ethylene, ABA, *Arabidopsis*

## Abstract

Ethylene regulates many aspects of plant growth and development including seed germination, leaf senescence, and fruit ripening, and of plant responses to environmental stimuli including both biotic and abiotic stresses. Ethylene response factors (ERFs) are plant-specific transcription factors and are a subfamily of the AP2 (APETALA2)/ERF transcription factor family. The function of many members in this large gene family remains largely unknown. ERF96, a member of the Group IX ERF family transcription factors, has recently been shown to be a transcriptional activator that is involved in plant defense response in *Arabidopsis*. Here we provide evidence that ERF96 is a positive regulator of abscisic acid (ABA) responses. Bioinformatics analysis indicated that there are a total four small ERFs in *Arabidopsis* including ERF95, ERF96, ERF97, and ERF98, and that ERF96 forms a cluster with ERF95 and ERF97. By using quantitative RT-PCR, we found that *ERF96* is expressed in all tissues and organs examined except roots, with relatively high expression in flowers and seeds. Results from the protoplast transfection assay indicated that the EDLL motif-containing C-terminal domain is responsible for ERF96’s transcriptional activity. Although loss-of-function mutant of *ERF96* was morphologically similar to wild type plants, transgenic plants overexpressing *ERF96* had smaller rosette size and were delayed in flowering time. In ABA sensitivity assays, we found that *ERF96* overexpression plants were hypersensitive to ABA in terms of ABA inhibition of seed germination, early seedling development and root elongation. Consistent with these observations, elevated transcript levels of some ABA-responsive genes including *RD29A*, *ABI5*, *ABF3*, *ABF4*, *P5CS*, and *COR15A* were observed in the transgenic plants in the presence of ABA. However, in the absence of ABA treatment, the transcript levels of these ABA-responsive genes remained largely unchanged. Our experiments also showed that water loss in *ERF96* overexpression plants was slower than that in Col wild type plants. Stomatal closure assays indicated that *ERF96* overexpression plants had reduced stomatal aperture in the presence of ABA. Taken together, our results suggest that ERF96 positively regulates ABA responses in *Arabidopsis*.

## Introduction

Ethylene response factors (ERFs) are a subfamily of the AP2 (APETALA2)/ERF superfamily, one of the several plant-specific transcription factor families (Riechmann et al., 2000). According to the number and similarity of their DNA binding domains, AP2/ERF superfamily is classified into five subfamilies: ERF, AP2, dehydration-responsive element (DRE) binding protein, related to ABSCISIC ACID INSENSITIVE3 (ABI3)/VIVIPAROUS1 (VP1), and others (Sakuma et al., 2002). ERF proteins contain only one AP2/ERF domain (Riechmann et al., 2000; Sakuma et al., 2002; Licausi et al., 2013).

Ethylene response factors are involved in the regulation of plant growth and development, primary and secondary metabolism, and plant responses to environmental stimuli including biotic and abiotic stresses (Gutterson and Reuber, 2004; Nakano et al., 2006; Xu et al., 2011; Licausi et al., 2013; Mizoi et al., 2013; Müller and Munné-Bosch, 2015). To date, ERF transcription factors have been identified and characterized from a number of plant species such as *Arabidopsis* (Nakano et al., 2006), rice (Nakano et al., 2006; Sharoni et al., 2011; Rashid et al., 2012), cotton (Jin and Liu, 2008), poplar (Zhuang et al., 2008), soybean (Zhang et al., 2008), barley (Gil-Humanes et al., 2009), grape (Zhuang et al., 2009), maize (Zhuang et al., 2010), tomato (Sharma et al., 2010), apple (Zhuang et al., 2011), cucumber (Hu and Liu, 2011), wheat (Zhuang et al., 2011), kiwifruit (Yin et al., 2012), peach (Zhang et al., 2012a), plum (Du et al., 2012), castor bean (Xu et al., 2013), Chinese cabbage (Li et al., 2013; Song et al., 2013), *Medicago truncatula* (Zhang et al., 2013), sorghum (Yan et al., 2013), sweet orange (Ito et al., 2014), and potato (Charfeddine et al., 2015).

In *Arabidopsis*, there are a total of 147 genes encoding AP2/ERF transcription factors, and 122 of them encode ERF transcription factors (Nakano et al., 2006). Based on phylogenetic analysis using the AP2/ERF domains, ERF transcription factors in *Arabidopsis* can be further classified into 12 different groups, namely, groups I to X, VI-L and Xb-L (Nakano et al., 2006).

Some of the group I and V ERF transcription factors have been shown to be involved in the regulation of the expression of lipids and cell wall components biosynthesis genes, basic type defense-related genes, pathogenesis-related genes, and osmotin, chitinase and β-1,3-glucanase encoding genes (Licausi et al., 2013). Some of them have been shown to be involved in the regulation of plant responses to abiotic and biotic stresses by either activating or repressing abscisic acid (ABA)-responsive genes (Gutterson and Reuber, 2004; Nakano et al., 2006; Xu et al., 2008, 2011; Licausi et al., 2013; Mizoi et al., 2013). For example, *AtERF4* over-expression plants were less sensitive to ABA inhibited root elongation which involves negative regulation of ethylene and ABA responses (Yang et al., 2005). AtERF7 binds to the GCC box and represses the expression of ABA-responsive genes (Zhang et al., 2007). ABR1 or ERF111 acts as a negative regulator of ABA responses during seed germination and ABA- and stress-regulated gene expression (Pandey et al., 2005) whereas transgenic plant overexpressing *AtERF13* confers ABA hypersensitivity in *Arabidopsis* (Lee et al., 2010). AtERF15 was shown to act as a positive regulator of ABA responses (Lee et al., 2015). On the other hand, ABA can also induce the expression of some ERF genes. For example, the expression of cotton ERF gene *GbERF*, tobacco ERF gene *NtCEF1* and tomato ERF gene *JERF1/3* has been shown to be induced by ABA (Wang et al., 2004; Zhang et al., 2004; Lee et al., 2005).

Subgroup IXc in group IX ERF subfamily contains four small ERFs with amino acids ranged from 131 to 139. These four ERFs are ERF95, ERF96, ERF97, and ERF98. In addition to the AP2/ERF domain, these ERFs contain an unknown function motif named CMIX-1 (Nakano et al., 2006). Among them, ERF95, also named ESE1 (ETHYLENE AND SALT INDUCIBLE 1), and ERF98 has been shown to be involved in the regulation of salt tolerance (Zhang et al., 2011, 2012b). ERF97, previously named AtERF14, has been shown to regulate plant defense response (Oñate-Sánchez et al., 2007). Recently, ERF96 has also been shown to regulate plant defense response (Catinot et al., 2015). Here we provide evidence that ERF96 is involved in the regulation of ABA response in *Arabidopsis*.

## Materials and Methods

### Plant Materials and Growth Conditions

*Arabidopsis* (*Arabidopsis thaliana*) ecotypic Columbia (Col-0) was used for protoplast isolation and plant transformation. The *Arabidopsis* mutant *erf96-1*, isolated from the transposon line GT_5_54244, is in *Landsberg erecta* (Ler) ecotypic background.

For seed germination, green seedlings, and root elongation assays, and for RNA isolation from seedlings, seeds were surface sterilized and sown on plates containing 0.6% (w/v) phytoagar solidified 1/2 Murashige and Skoog (MS) basal medium with vitamins (Plantmedia, Dublin, OH, USA) and 1% (w/v) sucrose. The plates were kept at 4°C in darkness for 2 days before being transferred to a growth room. For plant transformation and protoplasts isolation, seeds were sown directly into soil pots and grown in a growth room. The growth conditions in the growth room were set with temperature at 22°C, and photoperiod of 14 h light/10 h dark with light density of approximately 120 μmol m^–2^ s^–1^. For water loss and water-use efficiency assays, plants were grown in a growth chamber under a photoperiod of 10 h light/14 h dark photoperiod (short-day conditions).

### Plasmid Construction

To generate the *35S:HA-ERF96* and *35S:GD-ERF96* constructs, the full-length open-reading frame (ORF) of *ERF96* gene was amplified by RT-PCR using RNA isolated from 7-day-old *Arabidopsis* seedlings, and the PCR products were cloned in-frame with an N-terminal HA or GD tag into the *pUC19* vector under the control of the double *35S* enhancer promoter of *CaMV* (Wang et al., 2005). *35S:HA-ERF96* construct was digested with *Eco*R I restriction enzyme, and sub-cloned into the binary vector *pPZP211* for plant transformation (Hajdukiewicz et al., 1994).

To generate *35S:GD-EDLL* and *35S:GD-ERF96ΔEDLL* constructs, the nucleotide sequences encoding the EDLL motif containing C-terminal domain (amino acid 105–131) and *ERF96ΔEDLL* (amino acid 1–104) were amplified by RT-PCR using *35S:GD-ERF96* plasmids as template, and cloned in-frame with a GD tag into the *pUC19* vector. The primers used to amplify the full-length ORF of *ERF96* were 5′-ATGGATC-AAGGAGGTCGAGG-3′ and 5′-TCATTTCTTCTTGCCCTTG-3′. The primers used to amplify the *EDLL* motif were 5′-CATA-TGGAATTTGAGTACTTGGATG-3′ and 5′-TCATTTCTTCTT-GCCCTTG-3′. The primers used amplify the *ERF96ΔEDLL* were 5′-ATGGATCAAGGAGGTCGAGG-3′ and 5′-CTTAAGTC-AAAAAACTTGCCTAGAAG-3′.

### Plant Transformation and Transgenic Plant Selection

*Arabidopsis* plants of ∼5-week-old with several mature flowers on the main inflorescence were used for transformation via *Agrobacterium tumefaciens* GV3101-mediated floral dip method (Clough and Bent, 1998). T1 seeds were planted on 1/2 MS medium containing 50 μg/ml Kanamycin and 100 μg/ml Carbenicillin for selecting transgenic plants. Phenotypes of transgenic plants were examined in the T1 generation and confirmed in T2 up to T4 generations. Overexpression of *ERF96* in the transgenic plants was confirmed by RT-PCR. A minimum of five independent overexpression lines with similar phenotypes were obtained, and two homozygous lines were selected for further analysis.

### *Arabidopsis* Leaf Mesophyll Protoplast Transfection Assay

The procedure for *Arabidopsis* leaf mesophyll protoplast isolation, transfection and GUS activity assay had been described previously (Wang et al., 2005, 2007, 2014; Zhou et al., 2014; Liu et al., 2015). Plasmid DNAs for reporter and effector genes were isolated using the GoldHi EndoFree Plasmid Maxi Kit (Kangwei) according to the manufacturer’s instructions. GUS activities were measured by using a Synergy^™^ HT micro-plate reader (BioTEK).

### ABA Sensitivity Assays

For seed germination and green seedling assays, sterilized seeds were sown on 1/2 MS medium containing 0 (solvent alone), 1 or 2 μM ABA and grown in a growth room. Seed germination was scored 48 h after plates had been transferred into the growth room. Green seedlings were scored 10 days after transferring. The assays were repeated three times. For root elongation assay, 4-day-old seedlings grown on vertical plates were transferred to 1/2 MS medium plates containing 0 or 5.0 μM ABA and the plates were placed vertically. Root length was measured 6 days after seedling transferring. A minimum of 10 seedlings per line were used. For ABA-responsive gene expression assay, 7-day-old wild type and *ERF96* transgenic seedlings grown on vertical plates were transferred into 1/2 MS liquid medium without phytoagar and incubated for 90 min, then treated with 50 μM ABA for 2 h before the seedlings were frozen in liquid N_2_.

### RNA Isolation, RT-PCR, and Quantitative RT-PCR

Total RNA from *Arabidopsis* seedlings and different tissues and organs was isolated as described previously (Wang et al., 2014, 2015; Guo et al., 2015; Liu et al., 2015). cDNA was synthesized using 1 μg of total RNA by Oligo(dT)-primed reverse transcription using OMNISCRIPT RT Kit (QIAGEN). Quantitative RT-PCR (qRT-PCR) was used to examine the expression of ABA-responsive genes including *RESPONSIVE DROUGHT 29A* (*RD29A*), *ABSCISIC ACID INSENSITIVE 5* (*ABI5*), *ABSCISIC ACID RESPONSIVE ELEMENT-BINDING FACTOR 3* (*ABF3*), *ABRE BINDING FACTOR 4* (*ABF4*), *Δ^1^-PYRROLINE-5-CARBOXYLATE SYNTHETASE* (*P5CS*) and *COLD-RESPONSIVE 15A* (*COR15A*). The expression of *ACTIN2* (*ACT2*) was used as a control. qRT-PCR was performed on the Applied Biosystems 7500 real time PCR System using SYBR Green/ROX Master Mix (Thermo Scientific). The primers used for qRT-PCR examination of *RD29A*, *ABF3*, *P5CS*, *COR15A*, and *ACT2* have been described previously (Strizhov et al., 1997; Nakashima et al., 2006; Liu et al., 2014, 2015; Lu et al., 2015; Wang et al., 2015). Other primers used for qRT-PCR were: *ABI5*, 5′-GGAGATTGCGGACATTG-ATGAG-3′ and 5′-GGGAACACTAGTAAAGCAGATC-3′, *ABF4*, 5′-ACTGGAAGCCGAAATTGAAAAGCTC-3′ and 5′-CACCATGGTCCGGTTAATGTCCT-3′, *ERF95*, 5′-CCAT-TCTCAATTTTCCTCAC-3′ and 5′-AACTCAATAACTTCC-CTCCC-3′, *ERF96*, 5′-GCGGCTAGAGCCTATG-3′ and 5′-GTACTTG GATGATAGTG-3′, *ERF97*, 5′-ACCGTGGAGT-AAGGAGAC-3′ and 5′-GAAGTTGAGAATGGCAGC-3′, *ERF98*, 5′-GGAGCAGCAACAACCAAT-3′ and 5′-AGCGAG-ATGACCCCTAAG-3′.

### Water Loss and Stomatal Aperture Assays

Water loss was measured according to the method described by Tian et al. (2004). Briefly, rosettes of 5-week-old Col and *ERF96* transgenic plants were cut and weighed at different time points after cutting. The experiments were performed at room temperature under dim light conditions with 50% relative humidity. Three plants per genotype were used, and water loss was calculated as the percentage of initial fresh weight at each time point.

Stomatal aperture bioassay was performed as described by Lee et al. (2013) with some modifications. Four rosette leaves were detached from 5-week-old plants and floated in stomatal opening solution (SOS: 50 mM KCl and 10 mM MES-KOH, pH 6.15, 10 μM CaCl_2_) in light for 2 h, then the solution was replaced with SOS containing 20 μM ABA. Leaves were incubated for another 2 h before stomata were observed. For each sample, 100 stomatas were randomly observed under a digital microscope (eclipse 80i, Nikon Instruments Inc., Shanghai, China) and the width of individual stomata was recorded using Image J^1^. The experiment was repeated three times.

### Analysis of Water-Use Efficiency

About 5-week-old plants grown under short day conditions were sprayed with 75 μmol ABA or solvent containing distilled water as control. Instantaneous leaf water-use efficiency was measured 3 h after spraying by using a portable open-flow gas exchange system LI-6400 (LI-COR Biosciences, Lincoln, NE, USA), and calculated as described by Han et al. (2013).

## Results

### ERF96 is a Small ERF Transcription Factor

**FIGURE 1:**
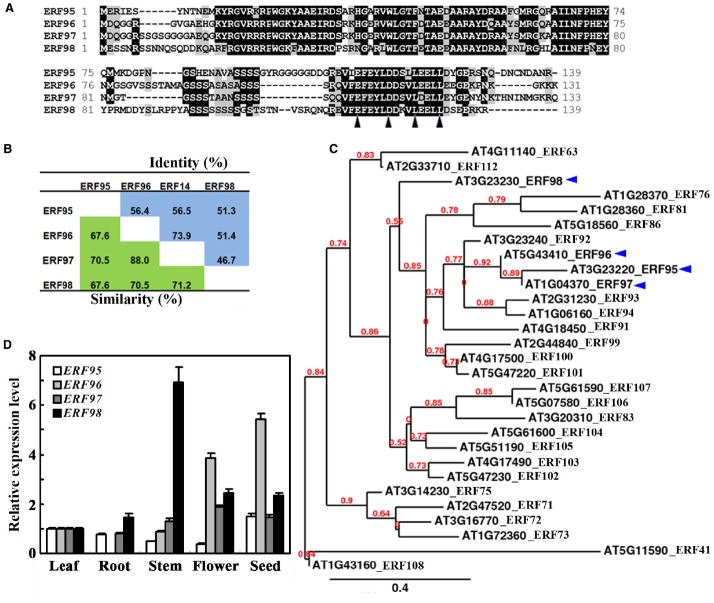
**Small ERFs in ***Arabidopsis***. (A)** Amino acid sequence alignment of small ERF proteins in *Arabidopsis*. Identical amino acids were shaded in black, and similar amino acids were in grey. Arrowheads indicate the conserved amino acid residues in the EDLL motif. **(B)** Amino acid similarity and identity of small ERF proteins in *Arabidopsis*. **(C)** Phylogenetic analysis of ERF96 and its homologues in *Arabidopsis*. **(D)** Expression pattern of *ERF95*, *ERF96*, *ERF97*, and *ERF98*. RNA was isolated from different tissues and organs of Col wide-type plants. Leaves and roots were from 4-week-old plants. qRT-PCR was used to examine the expression of the small *ERF* genes. The expression of *ACT2* was used as a control. The transcript level of the *ERF* genes in leaves was set at 1. Data represent mean ± SD of three biological replicates.

It has been reported that ERF96 belongs to the Group IX ERF family proteins (Nakano et al., 2006). In *Arabidopsis*, ERF95, ERF96, ERF97, and ERF98 are the only four small ERF proteins with amino acids ranged from 131 to 139 (Figure 1A). These four ERFs share 68–88% similarity and 47–74% identity at the amino acid level (Figure 1B). When the full-length amino acid sequence of ERF96 was used as a template to search for sequence homologues encoded by the *Arabidopsis* genome using the “Protein Homologs” search tool at Phytozome^2^, a total of 28 proteins were identified, and all of them are ERF proteins (Nakano et al., 2006). Phylogenetic analysis indicated that ERF95, ERF96, and ERF97 were closely clustered, whereas ERF98 was separated from this cluster (Figure 1C).

### Expression Patterns of *ERF96*

Previous experiments have shown that ERF95 and ERF98 are involved in the regulation of plant response to abiotic stresses (Zhang et al., 2012b), whereas ERF96 and ERF97 are involved in the regulation of plant response to biotic stresses (Oñate-Sánchez et al., 2007; Catinot et al., 2015). Because some of the ERFs have been shown to regulate plant responses to abiotic and biotic stresses by regulating ABA-responsive genes (Gutterson and Reuber, 2004; Nakano et al., 2006; Xu et al., 2008, 2011; Licausi et al., 2013; Mizoi et al., 2013), we wanted to examine whether small ERFs may involve in the regulation of ABA signaling in *Arabidopsis* by taking ERF96 as an example. To do that, we first examined the expression pattern of *ERF96* in *Arabidopsis* by using qRT-PCR. As shown in Figure 1D, relatively high expression of *ERF96* was observed in seeds and flowers whereas the transcript of *ERF96* in roots was undetectable. For comparison, we also examined the expression pattern of the other three small *ERF* genes. We found that none of them had an expression pattern similar to that of *ERF96* (Figure 1D).

### EDLL Motif is Responsible for ERF96’s Transcriptional Activity

**FIGURE 2:**
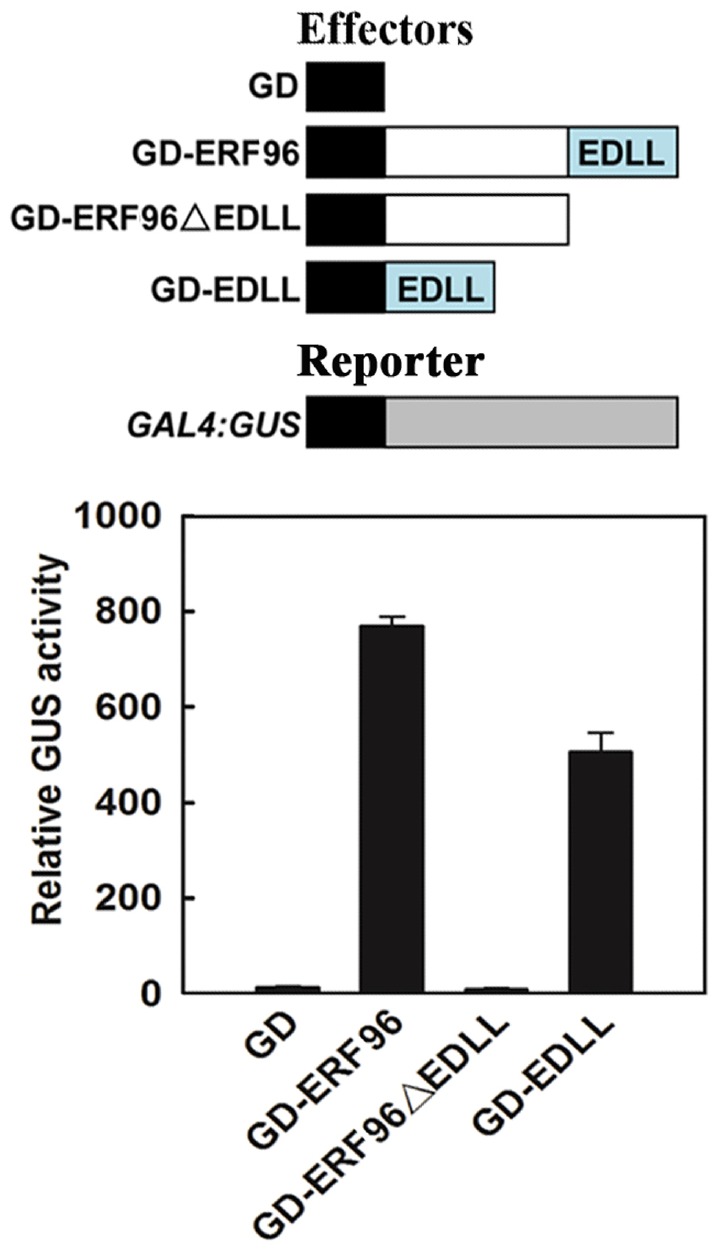
**The EDLL motif is responsible for ERF96’s transcriptional activity.** Plasmids of *GD-ERF96, GD-ERF96ΔEDLL* and *GD-EDLL* or *GD* alone (as a control) were co-transfected with a *GAL4:GUS* reporter into protoplasts isolated from rosette leaves of Col wild type plants. Transfected protoplasts were incubated in the darkness for 20–22 h before GUS activities were measured. Data represent mean ± SD of three replicates. Effectors and reporter used were diagrammed on the top of the figure.

Based on amino acid sequence analysis, small ERFs including ERF96 do not contain an obvious activation or repression domain. However, ERF96 functions as a transcription activator in protoplasts transfection assays (Catinot et al., 2015), and it contains an EDLL motif, a motif that has been shown to be presented in several ERF transcriptional activators (Tiwari et al., 2012), at its C-terminus (Figure 1A). We thus wanted to examine whether the EDLL motif is responsible for ERF96’s transcriptional activity by using the *Arabidopsis* mesophyll protoplast transient expression system (Wang et al., 2007). Plasmids of GAL4 DNA binding domain (GD) fused with ERF96, EDLL containing C-terminal domain (EDLL) or ERF096ΔEDLL were cotransfected with *GAL4:GUS* reporter plasmids into protoplasts. Cotransfection of *GD* plasmids was used as a control. As shown in Figure 2, both ERF96 and EDLL activated the expression of the reporter gene whereas ERF96ΔEDLL failed to do so, suggesting that the EDLL motif is responsible for ERF96’s transcriptional activity.

### Overexpression of *ERF96* Affects Plant Growth and Development

**FIGURE 3:**
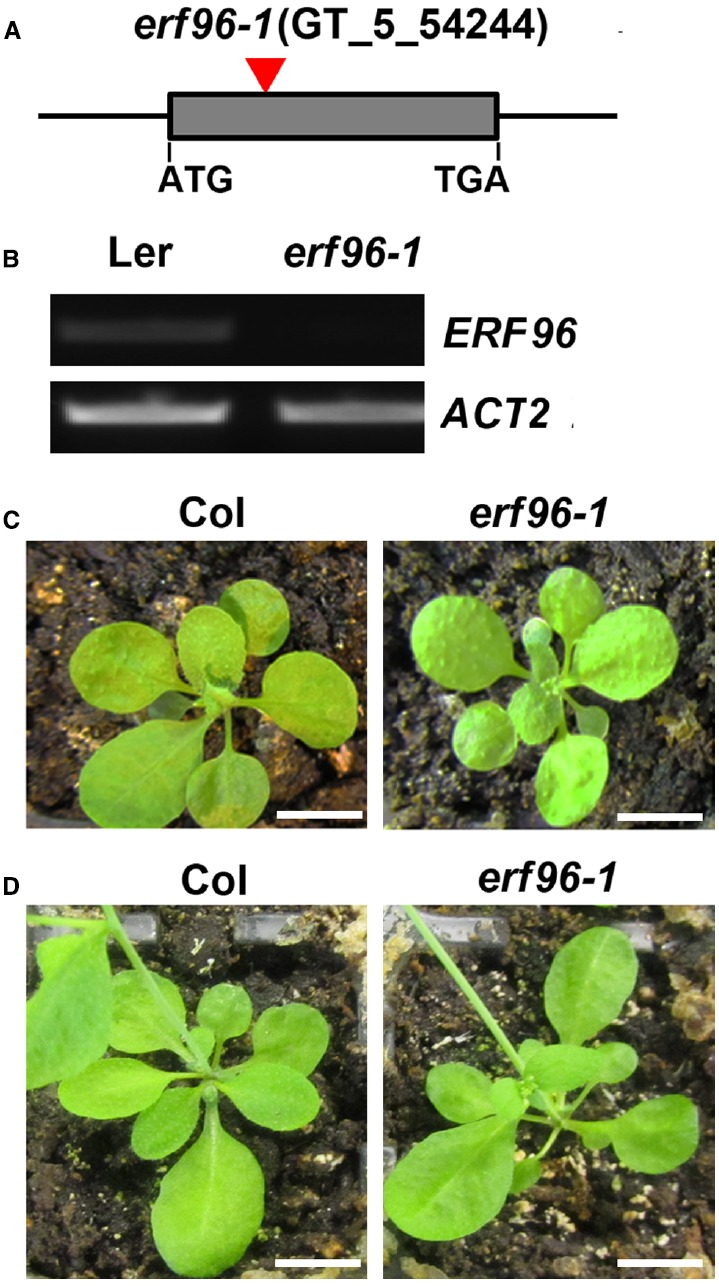
**Identification of the ***erf96-1*** mutant. (A)** Transposon insertion site in the *erf96-1* mutant. **(B)** RT-PCR analysis of *ERF96* transcript. RNA was isolated from 7-day-old seedlings. Expression of *ACT2* was used as a control. **(C)** The *erf96-1* mutant at vegetative stage. **(D)** The *erf96-1* mutant at reproductive stage. Bar in **(C,D)**, 1 cm.

**FIGURE 4:**
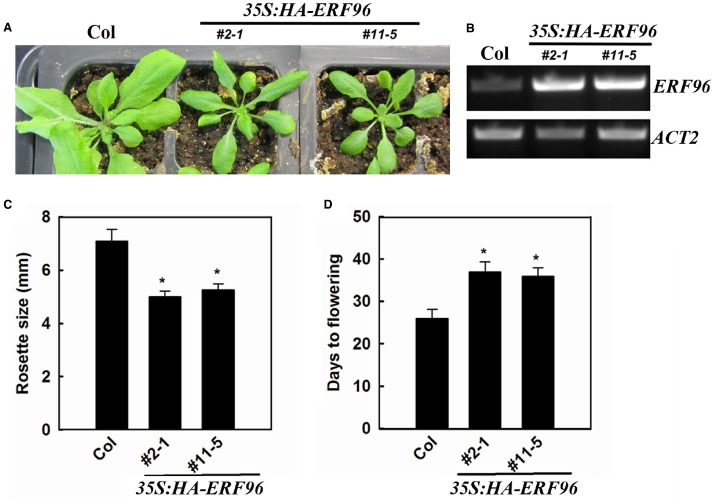
**Phenotypes of transgenic plants overexpressing ***ERF96***. (A)** Phenotypes of 4-week old *ERF96* overexpression plants. **(B)** Expression of *ERF96* in the transgenic plants. RNA was isolated from 7-day-old seedlings, and RT-PCR was used to examine the expression of *ERF96*. Expression of *ACT2* was used as a control. **(C)** Rosette size of the *ERF96* overexpression plants. **(D)** Flowering time of the *ERF96* overexpression plants. Data in **(C,D)** represent the mean ± SD of 10 individual plants. Asterisk (*) indicates significantly different from Col wild-type (*p* < 0.05).

To functionally characterize ERF96 in *Arabidopsis*, we first tried to identify loss-of-function mutant of *ERF96*. We found one transposon insertion line (GT_5_54244) through T-DNA Express: *Arabidopsis* Gene Mapping Tool^3^. In this line, the transposon was inserted at the first exon of *ERF96* gene (Figure 3A). RT-PCR analysis indicated that the full-length *ERF96* transcript was absent in this line (Figure 3B), suggesting that it represents a loss-of-function allele. This allele was designated as *erf96-1*. The *erf96-1* mutant displayed wild-type morphology at both vegetative and reproductive stages (Figures 3C,D). We then generated transgenic lines overexpressing *ERF96* in Col-0 ecotypic background. Morphologically, *ERF96* overexpression plants had smaller rosette size when compared with Col wild-type (Figures 4A,C). *ERF96* overexpression plants also showed late flowering phenotypes (Figures 4A,D).

### Transgenic Plants Overexpressing *ERF96* are Hypersensitive to ABA

**FIGURE 5:**
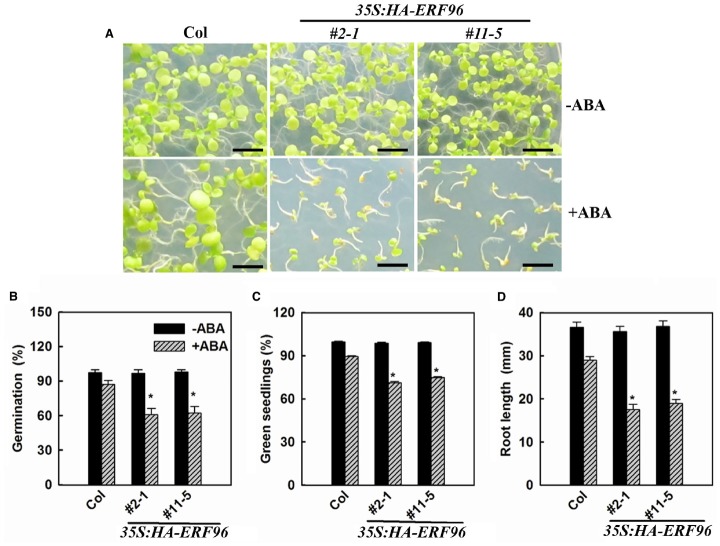
*****ERF96*** overexpression plants are hypersensitive to ABA. (A)** Ten-day-old Col wild type and *ERF96* overexpression seedlings grown on plates in the presence or absence of 2.0 μM ABA. Bar, 5 mm. **(B)** Effects of ABA on seed germination. Seeds were sown on plates in the presence or absence of 1.0 μM ABA, and percentage of seed germination was scored 48 h after the plates had been transferred into a growth room. **(C)** Effects of ABA on seedling greening. Seeds were sown on plates in the presence or absence 2.0 μM ABA. The percentage of green seedlings was scored 10 days after the plates had been transferred into a growth room. **(D)** Effects of ABA root elongation. The primary root length was measured 4 days after seedlings had been transferred to 1/2 MS plates containing 5.0 μM ABA. Data in **(B,C)** represent the mean ± SD of three replicates. Data in **(D)** represent the mean ± SD of 10 individual seedlings. Asterisk (*) indicates significantly different from Col in the presence of ABA (*p* < 0.05).

Some ERF proteins are involved in plant response to abiotic and biotic stresses (Gutterson and Reuber, 2004; Nakano et al., 2006; Xu et al., 2011; Licausi et al., 2013; Mizoi et al., 2013; Müller and Munné-Bosch, 2015). Available evidence suggested that this is also true for the small ERF proteins (Oñate-Sánchez et al., 2007; Zhang et al., 2011, 2012b; Catinot et al., 2015). Because ABA acts as one of the most important stress hormones and some ERFs such as AtERF4, AtERF7, and AtERF111 have been shown to be involved in ABA responses (Pandey et al., 2005; Yang et al., 2005; Zhang et al., 2007), we examined the ABA sensitivity of *ERF96* overexpression lines and the *erf96-1* mutant. We used the three different assays that have been commonly used to assess ABA sensitivities, including seed germination, early seedling development and root elongation. We found that in each of these assays, *ERF96* overexpression plants were hypersensitive to ABA (Figure 5), suggesting that ERF96 positively regulates ABA responses. However, *erf96-1* mutant had near wild-type responses in each of these assays (data not shown).

### Expression of Some ABA-Responsive Genes is Increased in Transgenic Plants Overexpressing *ERF96* Upon ABA Treatment

**FIGURE 6:**
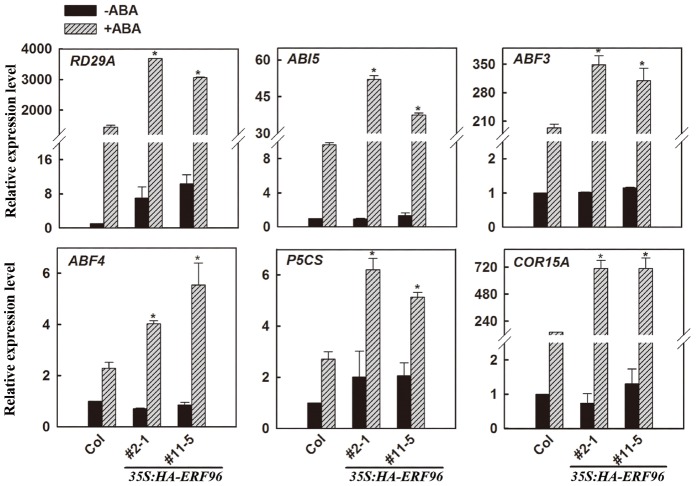
**Expression of ABA-responsive genes in *ERF96* overexpression plants.** RNA was isolated from 7-day-old seedlings with or without ABA treatment. qRT-PCR was used to examine the expression of *RD29A*, *ABI5*, *ABF3*, *ABF4*, *P5CS*, and *COR15A*. The expression of *ACT2* was used as a control. The transcript level of the corresponding gene in Col without ABA treatment was set at 1.0. Data represent the means ± SD of three biological replicates. Asterisk (*) indicates significantly different from that in ABA-treated Col wild type seedlings (*p* < 0.05).

Because the expression of *ERF96* was not induced by ABA (Hoth et al., 2002), and *ERF96* overexpression plants displayed ABA hypersensitivity in each of those three different assays (Figure 5), we wanted to further examine whether ERF96 may regulate the expression of ABA-responsive genes. Because *erf96-1* mutant was morphologically similar to wild type (Figure 3), had a near wild type response to ABA, and *ERF96* RNAi plants displayed a wild type defense response (Catinot et al., 2015), our assays hereon focused on *ERF96* overexpression plants. qRT-PCR was used to examine the expression of several selected ABA-responsive genes including *RD29A*, *ABI5*, *ABF3*, *ABF4*, *P5CS*, and *COR15A* (Baker et al., 1994; Strizhov et al., 1997; Kang et al., 2002; Nakashima et al., 2006; Liu and Stone, 2010). As shown in Figure 6, when compared with that in Col wild type seedlings, the expression of all the genes examined, but *RD29A*, remained largely unchanged in the transgenic plant seedlings in the absence of ABA. ABA treatment induced the expression of all of these genes in Col wild type seedlings, and the expression of these ABA-responsive genes was further elevated in *ERF96* overexpression plant seedlings when compared with that in the Col wild-type seedlings (Figure 6). These results indicate that ERF96 positively regulates ABA-induced gene expression, suggesting that ERF96 positively regulates ABA responses.

### ERF96 Regulates Water Loss

**FIGURE 7:**
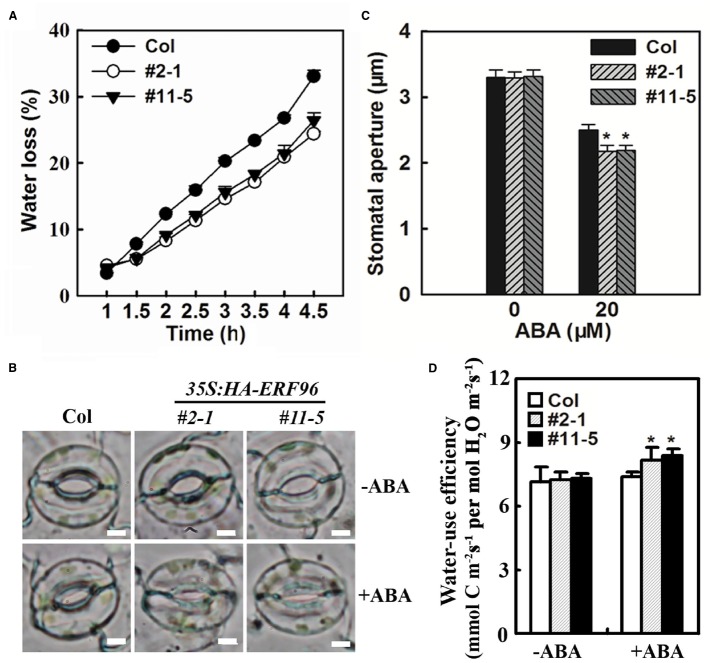
**Water loss and stomatal aperture in ***ERF96*** overexpression plants. (A)** Water loss assay in Col wild type plants and ERF96 overexpression plants. Whole rosettes of 5-week-old plants grown under short-day conditions were cut off from the base and used for water loss assay. Data represent the mean ± SD of three replicates. **(B)** Stomatal aperture in Col wild and *ERF96* overexpression plants. Shown are representative images of stomata before or after ABA treatment. Bar, 4 μm. **(C)** Measurement of stomatal apertures in Col wild and *ERF96* overexpression plants. Data represent the means ± SD of 100 stomata. Asterisk (*) indicates significantly different from that in Col before ABA treatment (*p* < 0.05). **(D)** Instantaneous leaf water-use efficiency in Col wild and *ERF96* overexpression plants. Data represent the means ± SD of three replicates. Asterisk (*) indicates significantly different from that in Col before ABA treatment (*p* < 0.05).

Abscisic acid is a critical regulator of stomatal movements that are associated with water loss in plants. Having shown that ERF96 functions as a positive regulator of ABA responses, we wanted to further examine whether *ERF96* overexpression plants display altered water loss. The water loss from the detached whole rosette of Col wild-type and *ERF96* transgenic plants grown in short-day conditions was measured. As shown in Figure 7A, *ERF96* overexpression plants lost water significantly slower than the Col wild-type plants. Consistent with these results, stomatal closure assays indicated that stomatal aperture in *ERF96* overexpression plants was small than wild type in the presence of ABA (Figures 7B,C). We also examined water-use efficiency in the *ERF96* transgenic plants. We found that, in the absence of ABA treatment, instantaneous leaf water-use efficiency in the *ERF96* transgenic plants was similar to that in the Col wild-type plants (Figure 7D). In the presence of ABA treatment, however, the instantaneous leaf water-use efficiency in the *ERF96* transgenic plants was higher when compared to that in the Col wild-type plants (Figure 7D).

## Discussion

ERF96 has recently been shown to regulate plant defense response (Catinot et al., 2015). In this study, we provide molecular and genetic evidence that ERF96 is a positive regulator of ABA responses.

### ERF96 is Involved in the Regulation of ABA Signaling

Among the small ERFs, ERF95 enhances transcript levels of salt-related genes, such as *COR15A*, *RD29A*, *P5CS2*, and *HOOKLESS1* (*HLS1*, Zhang et al., 2011). ERF97 is able to activate the transcription of some ERF-target genes including *PLANT DEFENSIN1.2* (*PDF1.2*) and *BASIC CHITINASE* (*CHIB*, Oñate-Sánchez et al., 2007), possibly through binding of the GCC box, which has been shown to be a binding site for several other *Arabidopsis* ERFs (Fujimoto et al., 2000). ERF98 promotes the expression of genes related to the AsA-GSH cycle, such as *ASCORBATE PEROXIDASE 3* (*APX3*), *APX6*, *CHLOROPLASTIC DHAR* (*ChlDHAR*), *CYTOSOLIC DHAR* (*CytDHAR*), and *GLUTATHIONE REDUCTASE 1* (*GR1*, Zhang et al., 2012b). ERF96 functions as a transcriptional activator, and it can directly activate some of the jasmonic acid/ethylene-response defense genes including *PDF1.2a*, *PATHOGENESIS-RELATED 3* (*PR-3*), *PR-4*, and *VEGETATIVE STORAGE PROTEIN 2* (*VSP2*; Catinot et al., 2015).

We provided evidence that ERF96 functions as a positive regulator of ABA responses (Figure 5). Expression of some ABA-responsive genes including *RD29A*, *ABI5*, *ABF3*, *ABF4*, *P5CS*, and *COR15A* was elevated upon ABA treatment (Figure 6). However, considering the fact that the expression of some ABA-responsive genes remained largely unchanged in the *ERF96* overexpression plants in the absence of ABA (Figure 6), it is unlikely that ERF96 directly activates the expression of these ABA-responsive genes. Further studies will be required to pinpoint the precise action site of ERF96 in the ABA signaling network.

### ERF96 May Function Redundantly with Other ERFs to Regulate Plant Growth and ABA Responses

Functional redundancy has been a common theme for members of ERF transcription factors. So far, phenotypes have only been observed in loss-of-function alleles of a very limited number of ERFs, such as the loss-of-function mutants of *AtERF4* and *NICOTIANA BENTHAMIANA CELL DEATH* (*NbCD1*, McGrath et al., 2005; Nasir et al., 2005). The absence of scorable phenotypes in null alleles of most *ERFs* has made it difficult to assess the function of ERFs through loss-of-function studies. Although overexpression study has its limitation in defining gene function, it has been helpful for the characterization of some ERFs. For example, overexpression of *AtERF7* reduced ABA responses in guard cells and decreased drought tolerance (Song et al., 2005), overexpression of *CYTOKININ RESPONSE FACTOR 5* (*CRF5*) increased pathogen resistance and activated the expression of a large number of GCC-box pathogenesis-related genes (Liang et al., 2010), and overexpression of *TRANSLUCENT GREEN* (*TG*) in transgenic plants conferred enhanced drought tolerance (Zhu et al., 2014). Plants overexpressing *ERF96* showed enhanced defense response, however, *ERF96* RNAi plants had wild type response (Catinot et al., 2015). We found that loss-of-function allele of *ERF96* did not show any morphological phenotypes and had a near wild-type ABA sensitivity, whereas *ERF96* overexpression plants displayed morphological phenotypes and showed hypersensitivity to ABA (Figures 3–5). Thus it is likely that ERF96 may function redundantly with other ERFs including small ERFs. Combination of double, triple and higher order mutations in ERFs may help address the functional redundancy of ERFs.

In summary, we provide molecular, biochemical and genetic evidence that ERF96 functions as a positive regulator of ABA responses. Overexpression of *ERF96* also conferred reduction in water loss from leaf surface likely through more sensitive stomatal closure. Manipulating the expression level of *ERF96* may help improve crops’ water use efficiency in agriculture.

## Author Contributions

SW and JC conceived the study. XW, SW, and JC designed the experiments. XW, SL, and HT performed the experiments. XW, SW, and JC analyzed the data. XW drafted the manuscript, and all authors read and approved the final manuscript.

### Conflict of Interest Statement

The authors declare that the research was conducted in the absence of any commercial or financial relationships that could be construed as a potential conflict of interest.

## Footnotes

1 http://imagej.nih.gov/ij

2 http://phytozome.jgi.doe.gov/pz/portal.html

3 http://signal.salk.edu/cgi-bin/tdnaexpress
